# Supplementary material for: Single spin localization and manipulation in graphene open-shell nanostructures
Source: Nat Commun. 2019 Jan 14;10:200. doi: 10.1038/s41467-018-08060-6 (PMC6331630; doi:10.1038/s41467-018-08060-6)
Supplement: Supplementary file 1 — Supplementary Information [file 41467_2018_8060_MOESM1_ESM.pdf]

Supplementary Information for:
Single Spin Localization and Manipulation in Graphene Open-Shell Nanostructures

Jingcheng Li[1], Sofia Sanz[2], Martina Corso[2,3], Deung Jang Choi[2,3,5], Diego Peña[4], Thomas Frederiksen[2,5] & Jose Ignacio Pascual[1,5]

[1]*CIC nanoGUNE, 20018 Donostia-San Sebastián, Spain*
[2] *Donostia International Physics Center (DIPC), 20018 Donostia-San Sebastián, Spain*
[3]*Centro de Física de Materiales MPC (CSIC-UPV/EHU), 20018 Donostia-San Sebastián, Spain*
[4]*Centro Singular de Investigación en Química Biolóxica e Materiais Moleculares (CiQUS), and Departamento de Química Orgánica, Universidade de Santiago de Compostela, Spain*
[5]*Ikerbasque, Basque Foundation for Science, 48013 Bilbao, Spain* *December 7, 2018*

Contents

Reaction pathway for the formation of the GNR junctions **2**

Statistics of different types of junctions **3**

Temperature and magnetic field evolution of the Kondo resonance **4**

Understanding the appearance of spins in the junctions from Clar's theory **5**

Transport spectra of Type 2 junctions **6**

DFT simulations **7**

Mean-field Hubbard model **10**

Singlet-triplet excitations vs molecular size **19**

References **20**

Supplementary Note 1: Reaction pathway for the formation of the GNR junctions

Supplementary Figure 1 shows a plausible mechanism for the formation of the GNR junctions. First, CH activation would form the corresponding sigma-radicals which could evolve by C-C coupling followed by cyclodehydrogenation to obtain a five-membered ring (in red). Then, fragmentation of a benzene ring, followed by H migration could lead to a terminal alkyne (in blue). Finally, isomerization of a double bond, followed by cyclization of the terminal alkyne with a bay region of the GNR, could afford the final GNR junction.

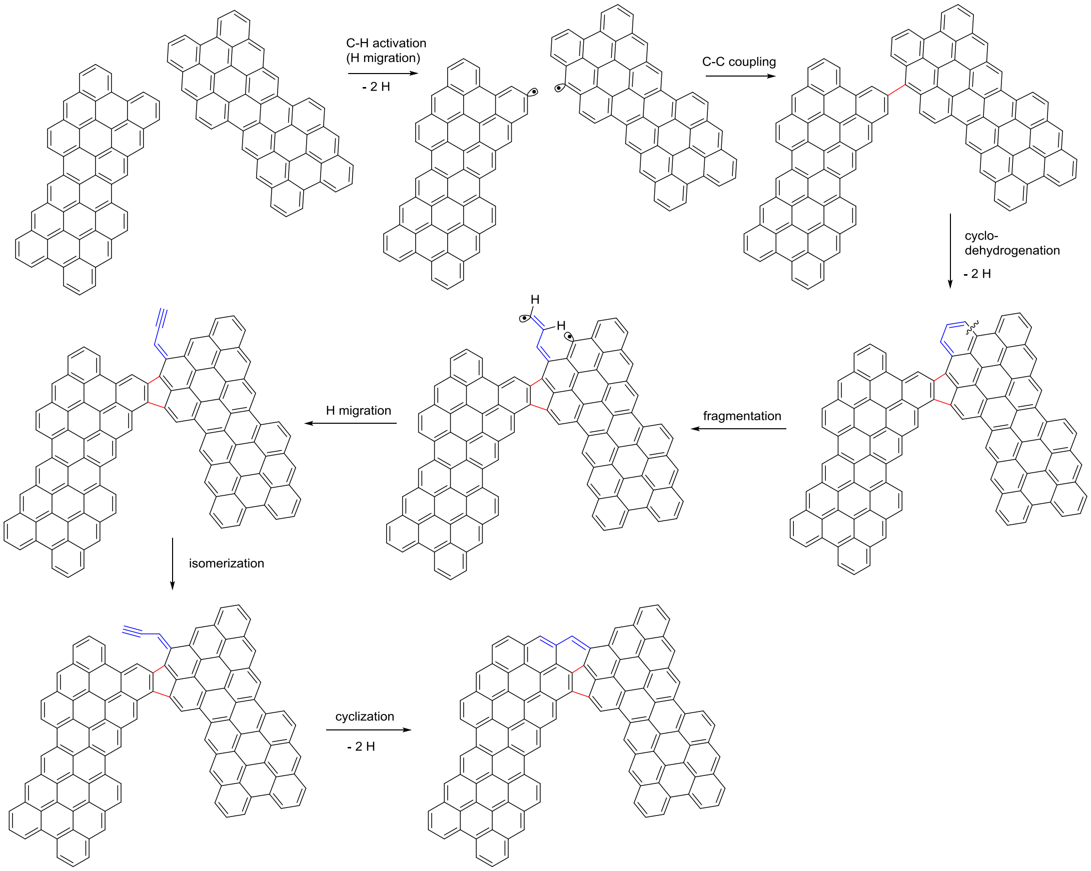

Supplementary Figure 1. Proposed reaction pathway for the formation of the graphene nanostructures.

Supplementary Note 2: Statistics of different types of junctions

In the main text we studied three different nanostructure junctions (Figs. 1 and 2). Here we show the frequency statistics of the three types. From the 45 different junctions studied, 30 of them are identified as Type 3 junctions, while 9 and 5 of them are identified as Type 1 and Type 2 junctions respectively (Supplementary Figure 2a). From this statistics, we deduced that the ZZ sites are more favorable to incorporate an extra hydrogen atom and get passivated, becoming Type 1 junctions (22% of the radicals). The PC sites had an extra atom only in 13% of the cases, thus appearing as Type 2 junctions. The overall percentage of H-passivation observed here is comparable to the value found at the termination of armchair GNRs[1], which happened in 15% of the occasions.

In one case out of the 45 nanostructures studied, the junction appeared with same backbone structure as the other, but with neither bright sites, nor zero-bias features in the spectra. Supplementary Figure 2b shows a constant height current image of that junction. The ring structure can be now nicely resolved, following the sketch in Fig. 1d of the main text. The absence of spins here is attributed to the passivation of both radical sites by two extra H atoms.

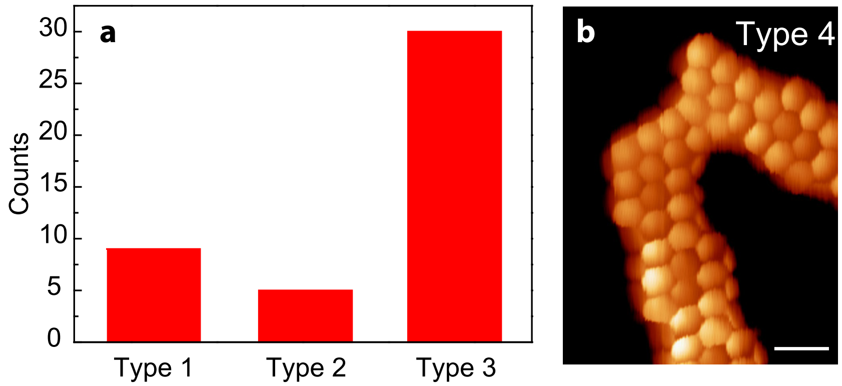

Supplementary Figure 2. Statistics of different types of junctions. a, Bar plot of the number of three types of junctions studied in the main text. b, Constant-height current image ($V = 2$ mV, scale bar 0.5 nm) shows another type of junctions with both radicals are passivated by H atoms, which is classified as Type 4 junction.

Supplementary Note 3: Temperature and magnetic field evolution of the Kondo resonance

Figure 2 in the main text shows the temperature evolution of the Kondo resonance (Figure 2c) and the broadening of its line-width at 1.2 K in response to an external applied magnetic field (Figure 2d). Both are fingerprints of the Kondo-origin of the zero-bias resonance, which signals the presence of a localized magnetic moment. The linewidth of the Kondo resonance was obtained from a Frota function [2], as indicated in ref. [3], and corrected by the thermal broadening of the tips Fermi edge using a simple quadratic approximation. We obtain that at the lowest temperature of our experiment (1.07 K) and smallest bias modulation (100 μeV) the intrinsic HWHM of the zero-bias resonance is 0.64 meV. Such narrow linewidth is unrealistic for a single-particle state, because it would imply a negligible interaction with the environment and, since it lies at zero bias - i.e. it is singly occupied, a negligible effect of Coulomb e-e blockade in the tunneling process.

Instead, we demonstrate in the main text (inset of Fig. 2c) that the HWHM follows an additional unconventional thermal broadening, after removal of the thermal broadening of the tips Fermi edge. This broadening follows the empirically expression $\frac{1}{2}\sqrt{(\alpha k_B T)^2 + (2k_B T_K)^2}$ obtained for Kondo resonances in the Fermi Liquid approximation [3,4], and we obtain a Kondo temperature $T_K \sim 6$ K and $\alpha = 9.5$. The narrow linewidth allows us to also explore the effect of an external magnetic field. As shown in Figure 2d and here, in Supplementary Figure 3, a significant reduction of intensity and a linewidth broadening amounting to $\sim$ 0.6 meV is detected for applied magnetic fields of 2.8 T, in agreement with the expected behaviour of a Kondo resonance caused by a spin 1/2. As shown in Ref. [5], for temperatures sufficiently below T_K, the Kondo resonance of a spin should split above a critical magnetic field of B $\sim$0.5 T_k. The flat cusp suggests that at the maximum B of our setup we are close to a complete split, which should then occur at $\sim$ 3 T.

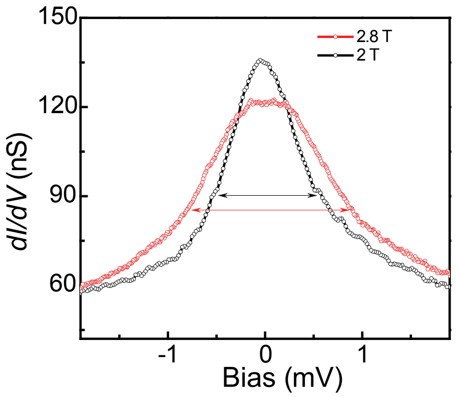

Supplementary Figure 3. Magnetid field broadening of the Kondo resonance. We compare here the zero-bias resonance presented in Fig. 2d for 0 T and 2.8 T. Both spectra are measured at 1.2K and with a bias modulation of 0.1 mV @ 760 Hz. The FWHM of resonance at 2.8 T is $\sim 2{\times}300$ μeV broader, in agreement with the expected broadening of a spin 1/2.

Supplementary Note 4: Understanding the appearance of spins in the junctions from Clar's theory

In the main text, we used both DFT and Hubbard Mean Field simulations to show that the spins in Type 3 junctions comes from two in-gap states, simply occupied as a result of electron-election correlations. An alternative and intuitive chemical picture behind the emergence of singly occupied radical states can be drawn bearing in mind Clar's aromatic pi-sextet rule [6]. Supplementary Figure 4 shows two possible resonance structures for the GNR junction: the closed shell structure a, with 8 Clar sextets, and the open shell structure b, with 11 Clar sextets and two radicals at the PC and ZZ sites. The dominance of resonance structure b in our experiments means that the energy required to create the two unpaired electrons (radicals) in structure b is compensated by the stabilization provided by the presence of three additional Clar sextets. In fact, the radical sites can delocalize towards the two second neighbor edge carbon atoms, agreeing with the carbon sites with high density of states shown in Fig. 1e in the main text. Hence, this phenomenological model can qualitatively explain the spontaneous appearance of spin in the nanostructures.

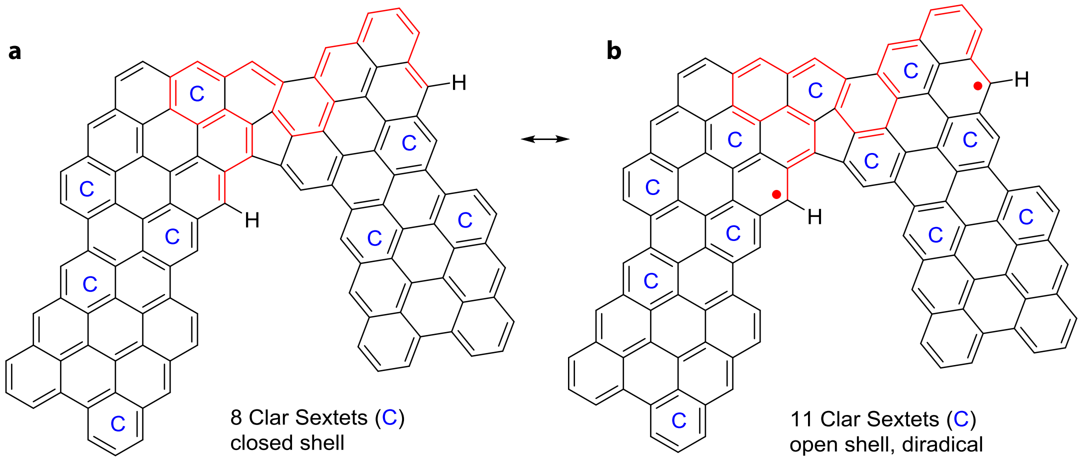

Supplementary Figure 4. Understanding the appearance of spins in the nanostructures from Clar's theory. a, Close-shell resonant structure of a Type 3 nanostructure, with its Clar's sextets indicated with "c". b, Di-radical form of the model structure in a, hosting three additional sextets. Only the H atom of the sites becoming radicals is shown in this model. Structure b is the energetically most stable if the energy gain by incorporating the additional sextets compensates the energy cost for creating the pair of radicals. In this case, the di-radical form is expected to show spin-polarization at the radical sites.

Supplementary Note 5: Transport spectra of Type 2 junctions

In Fig. 5 of the main text, we studied the behaviour of a spin localized at the PC site in a transport measurement, when a Type 3 junction was contacted with the STM tip at the neighbour ZZ site and lifted. Electrons injected through the conjugated backbone reproduced the Kondo resonance observed in tunneling regime. For comparison, here we show similar transport measurement for a Type 2 junction, i.e. when there is no spin in the graphene nanostructure. As in the other case, the STM tip was approached to the radical at the ZZ site to make a bond between nanostructure and STM tip (illustrated in Supplementary Figure 5). Before bond formation, the characteristic Kondo resonance of Type 2 junctions is observed in the dI/dV spectra (point 1 in the figure). However, once the radical bonded to the tip (signalled by the characteristic jump-to-contact step), the junction bridged tip and substrate and the Kondo resonance disappeared. This proves that the tip-radical contact quenches the magnetic moment of this site, as presumed Figure 5 of the main text. It also proves that the Kondo feature observed in Fig. 5 for the lifted junction correspond to the PC spin embedded in the cGNR junction.

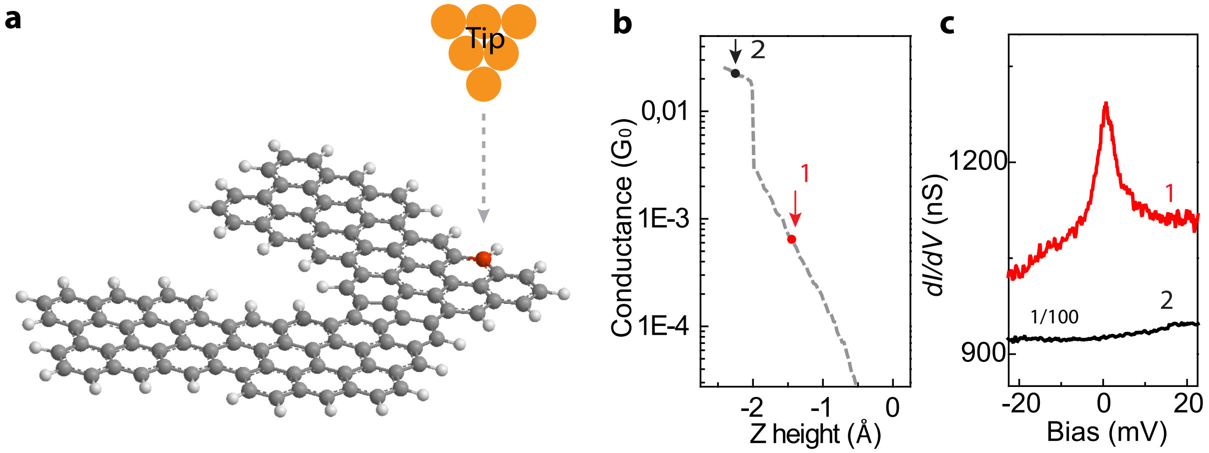

Supplementary Figure 5. Transport properties of the lifted junctions without radical. a, Schema illustrating the process, when the STM tip was approached to the ZZ radical of a Type 2 junction (gray dashed arrow) to form a contact. b, Simultaneously recorded conductance curve ($V = -50$ mV) during the process in a. Red and black dots indicate the vertical positions at whcih dI/dV spectra in c were taken.

Supplementary Note 6: DFT simulations

We performed calculations with the SIESTA implementation [7] of density functional theory (DFT). Exchange and correlation (XC) were included within either the local (spin) density approximation (LDA) [8] or the generalized gradient approximation (GGA) [9]. We used a 400 Ry cutoff for the real-space grid integrations and a double-zeta plus polarization (DZP) basis set generated with an 0.02 Ry energy shift for the cutoff radii. The molecules, represented with periodic unit cells, were separated by a vacuum of at least 10 Å in any direction. The electronic density was converged to a stringent criterion of 10^5. The force tolerance was set to 0.002 eV/Å. In Fig. 3 in the main text we report the GGA results.

Role of exchange-correlation functional In Supplementary Figure 6 we compare the calculated spin polarization for the generic (2,2) graphene nanojunction within both LDA [8] and GGA [9] XC approximations. We also compare the real-space spin density with a Mulliken population analysis. From Supplementary Figure 6 it is clear that the emerging picture for the radicals is robust among all four approaches. As expected, the intensity of the spin polarization is more pronounced in GGA than in LDA.

Energetically preferred hydrogen passivation sites In the main text we showed the hydrogen passivation on ZZ sites (Type 1 junctions) and PC sites (Type 2 junctions). In Supplementary Figure 2 we report a higher probability of hydrogen passivation on ZZ sites than PC sites. To quantitatively study this phenomenon, we analysed the energetics of different hydrogen passivation of the edges from DFT simulations. The results are summarized in Supplementary Figure 7. We find that hydrogen passivation on the ZZ and PC sites are indeed the two most stable configurations, with the former being the energetically most favoured one. This is in agreement with the experimental observations (Supplementary Figure 2).

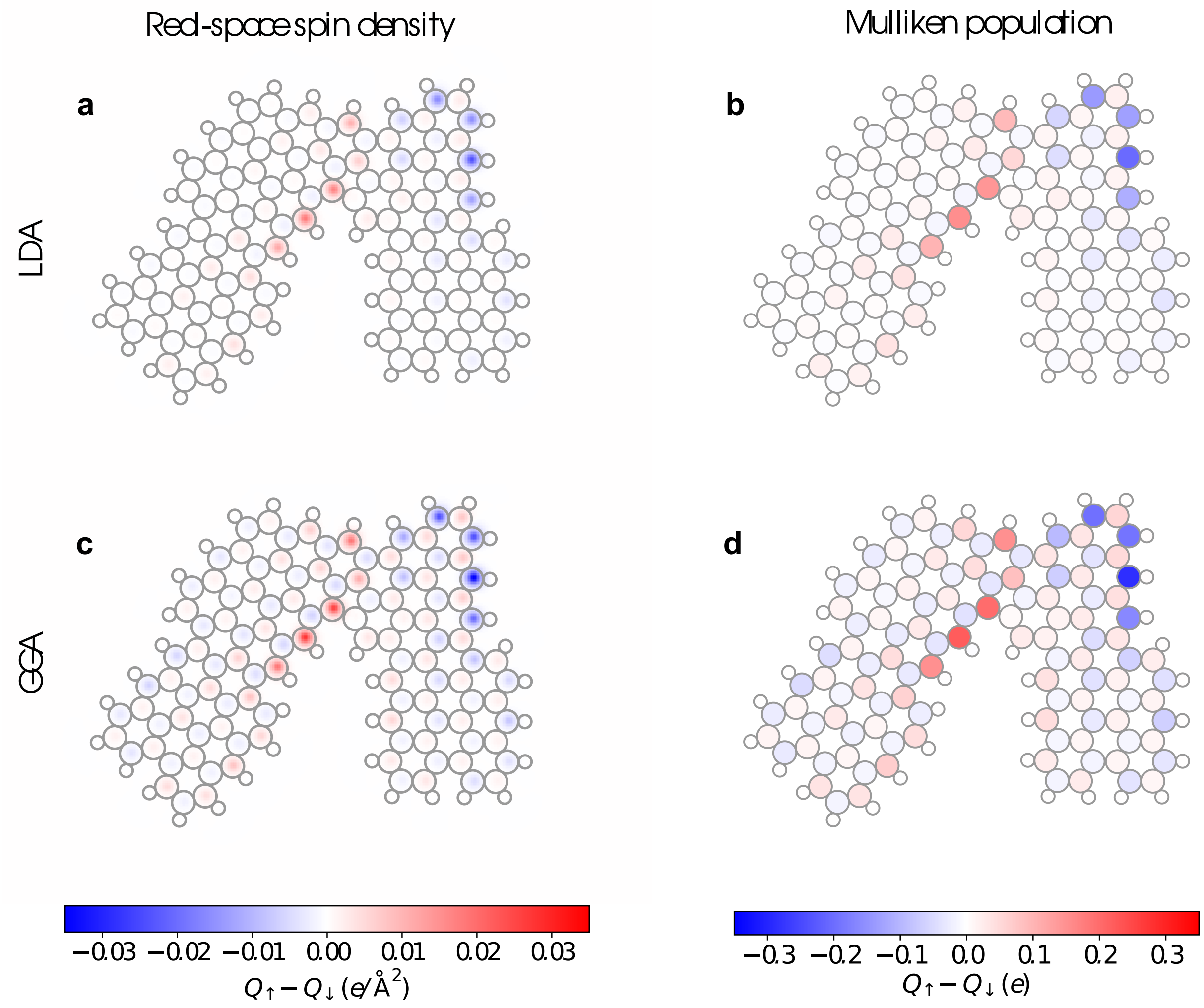

Supplementary Figure 6. Spin polarization in the (2,2)-junction from DFT simulations. a, Real-space spin density calculated within LDA. b, Mulliken population analysis of the spin density calculated within LDA. c,d Same as a,b but for GGA. Panel d corresponds to Fig. 3a in the main text.

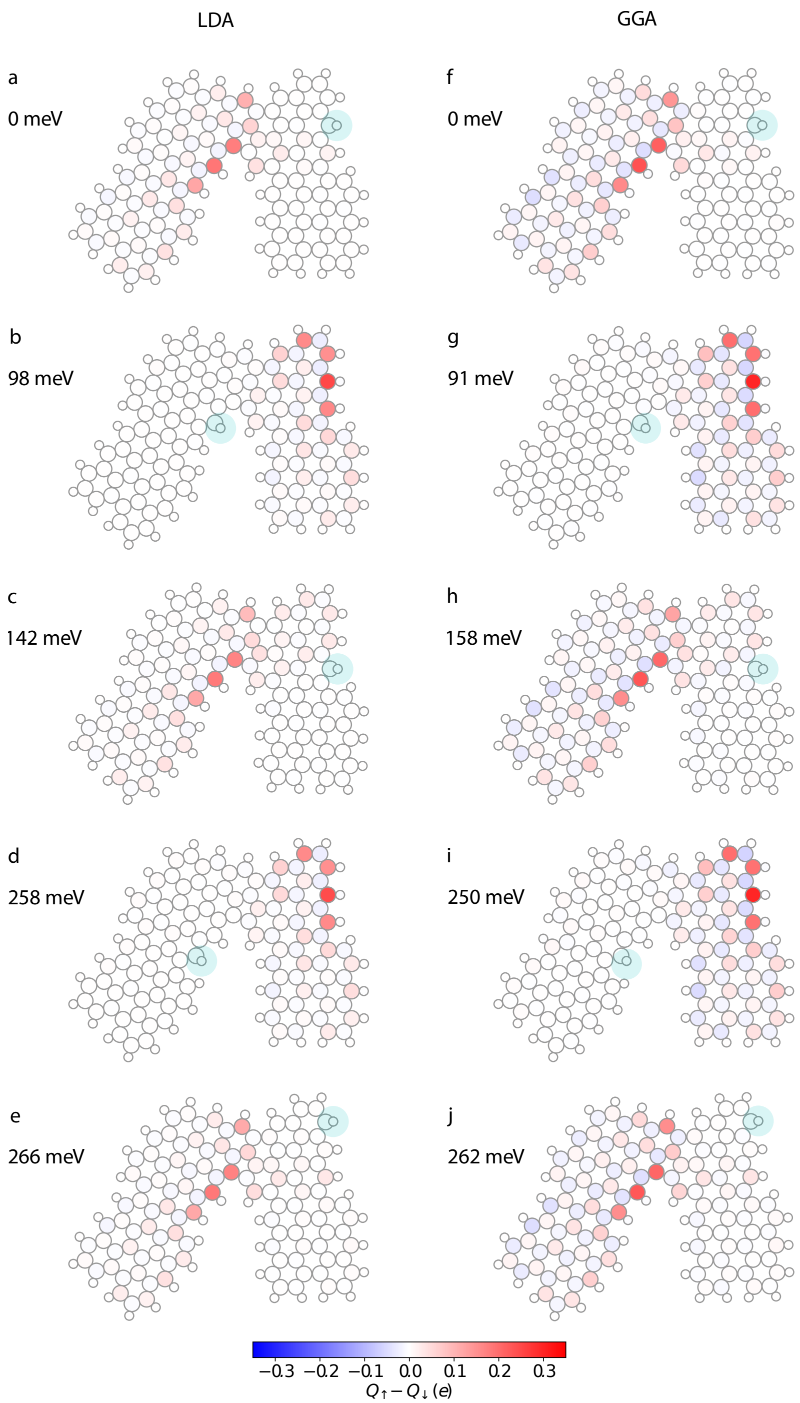

Supplementary Figure 7. Energetics and spin polarization for five energetically preferred hydrogen passivation sites from DFT simulations. a-e, Results from LDA calculations via a Mulliken population analysis. The hydrogen passivation (blue circles) shown in a on the ZZ site is the most stable configuration, while the other sites are less energetically favoured with the energy differences quoted in each panel. f-j, Same molecules as in a-e but results from GGA calculations. Panels f,g correspond to Fig. 3b in the main text.

Supplementary Note 7: Mean-field Hubbard model

To complement the DFT simulations described above we also performed simulations based on the mean-field Hubbard (MFH) model, known to provide a good description for carbon π-electron systems [10–14]. We describe the graphene nanostuctures with the following Hamiltonian for the sp^2 carbon atoms:

$$
\begin{aligned}
H &= -t_1 \sum_{\langle i,j\rangle,\sigma} (c^\dagger_{i\sigma} c_{j\sigma} + \text{h.c.}) - t_2 \sum_{\langle\langle i,j\rangle\rangle,\sigma} (c^\dagger_{i\sigma} c_{j\sigma} + \text{h.c.}) - t_3 \sum_{\langle\langle\langle i,j\rangle\rangle\rangle,\sigma} (c^\dagger_{i\sigma} c_{j\sigma} + \text{h.c.}) \\
& \quad + U \sum_{\langle i} (n_{i\uparrow}\langle n_{i\downarrow}\rangle + \langle n_{i\uparrow}\rangle n_{i\downarrow} - \langle n_{i\uparrow}\rangle\langle n_{i\downarrow}\rangle) \qquad (1)
\end{aligned}
$$

where $c_{i\sigma}$ ($c^\dagger_{i\sigma}$) annihilates (creates) an electron with spin σ in the p_z orbital centred at site i. The first three terms describe a tight-binding model with hopping amplitudes t_1, t_2, and t_3 for the first, second, and third-nearest neighbour matrix elements (defined in terms of interatomic distances $d_1 < 1.6$Å$< d_2 < 2.6$Å$< d_3 < 3.1$Å). We follow the parameterizations of Ref. [14] and consider both a simple first-nearest neighbour (1NN) model with $t_1 = 2.7$ eV and $t_2 = t_3 = 0$ as well as a more accurate third-nearest neighbour (3NN) model with $t_1 = 2.7$ eV, $t_2 = 0.2$ eV, and $t_3 = 0.18$ eV.

The term proportional to the empirical parameter U accounts for the on-site Coulomb repulsion. By comparison with first-principles simulations it has been established that DFT-GGA (DFT-LDA) are generally best reproduced when $U/t \approx 1.3$ (0.9) [12]. Consistent with this, we find a good overall agreement with our experimental observations using $U \sim 3.5$ eV as analysed below.

The expectation value of the spin-resolved density operator $n_{i\sigma} = c^\dagger_{i\sigma} c_{i\sigma}$ is computed from the eigenvectors of H. From the self-consistent solution of the Hamiltonian in Eq. (1) we obtain the local spin density from the charge difference $Q_{i\uparrow} - Q_{i\downarrow}$, with $Q_{i\sigma} = e\langle n_{i\sigma}\rangle$. In units of μ_B the magnetization is $M_i = (n_{i\uparrow} - n_{i\downarrow})/2$.

We solve the mean-field Hubbard model using a custom-made Python implementation based on SISL [15]. In Fig. 3 in the main text we report the non-interacting single-particle wave functions in the 3NN model ($U = 0$) as a basis to understand the open-shell electronic configurations obtained in DFT and MFH.

Band structure of infinite (3,1)cGNRs As shown in Supplementary Figures 8 and 9, both the first-neighbour and third-neighbour MFH models (red bands) provide a good description for the 1D band structure of the (3,1) chiral graphene nanoribbon (cGNR) as compared to DFT calculations (black bands) obtained with SIESTA [7]. Unlike DFT and the 3NN model, the simple 1NN model implies electron-hole symmetry of the bands. The low-energy part of the DFT band structure is generally very well reproduced with MFH using an on-site Coulomb repulsion of $U \leq 3.5$ eV. Indeed this narrow cGNR is intrinsically non-magnetic, consistent with previous works [12,13,16].

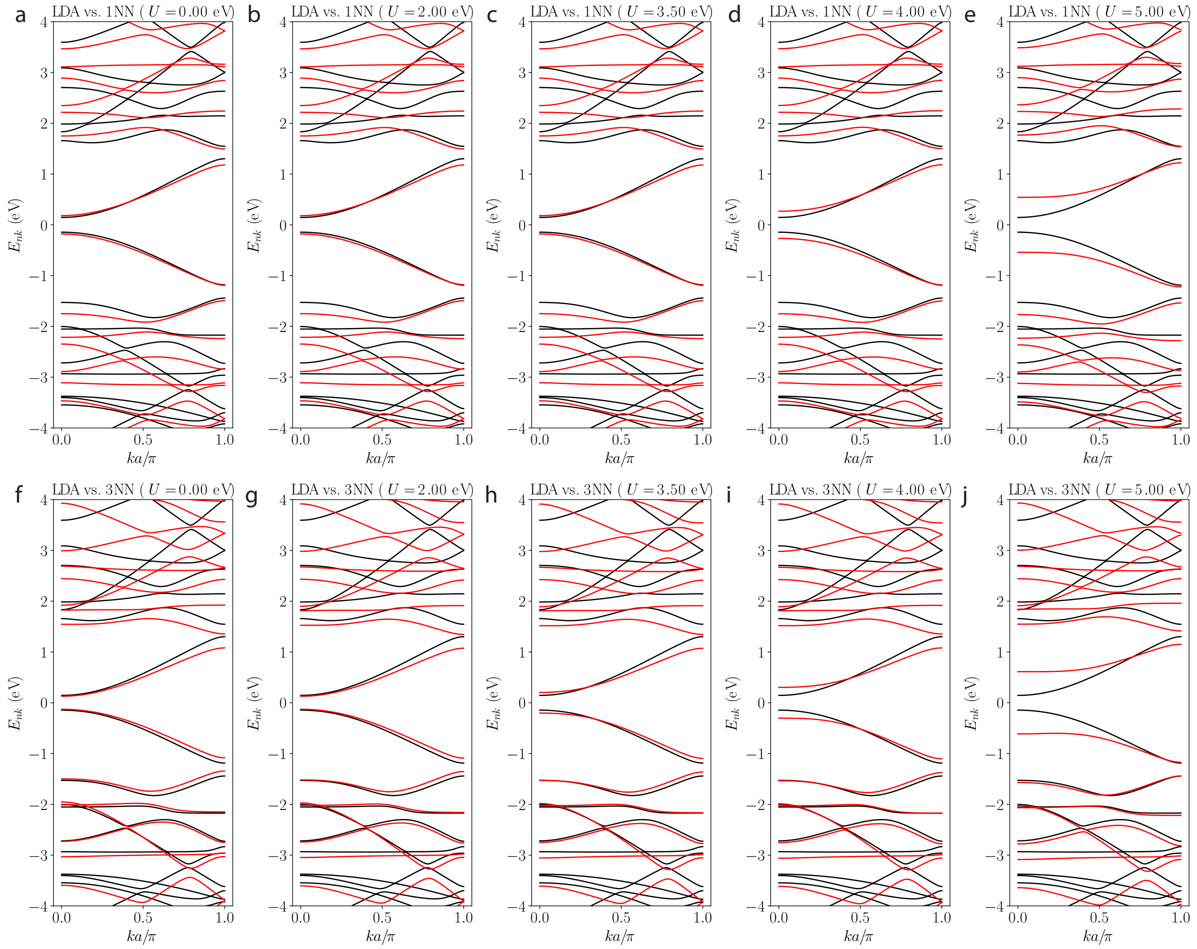

Supplementary Figure 8. Calculated 1D band structure for the (3,1)cGNR. We compare DFT-LDA (black lines) with the mean-field Hubbard model with different Coulomb repulsion U (red lines) within either (**a-e**) first-nearest neighbour couplings only ($t_1 = 2.70$ eV, top row) or (**f-j**) third-nearest neighbour couplings ($t_1 = 2.70$ eV, $t_2 = 0.20$ eV, and $t_3 = 0.18$ eV, bottom row).

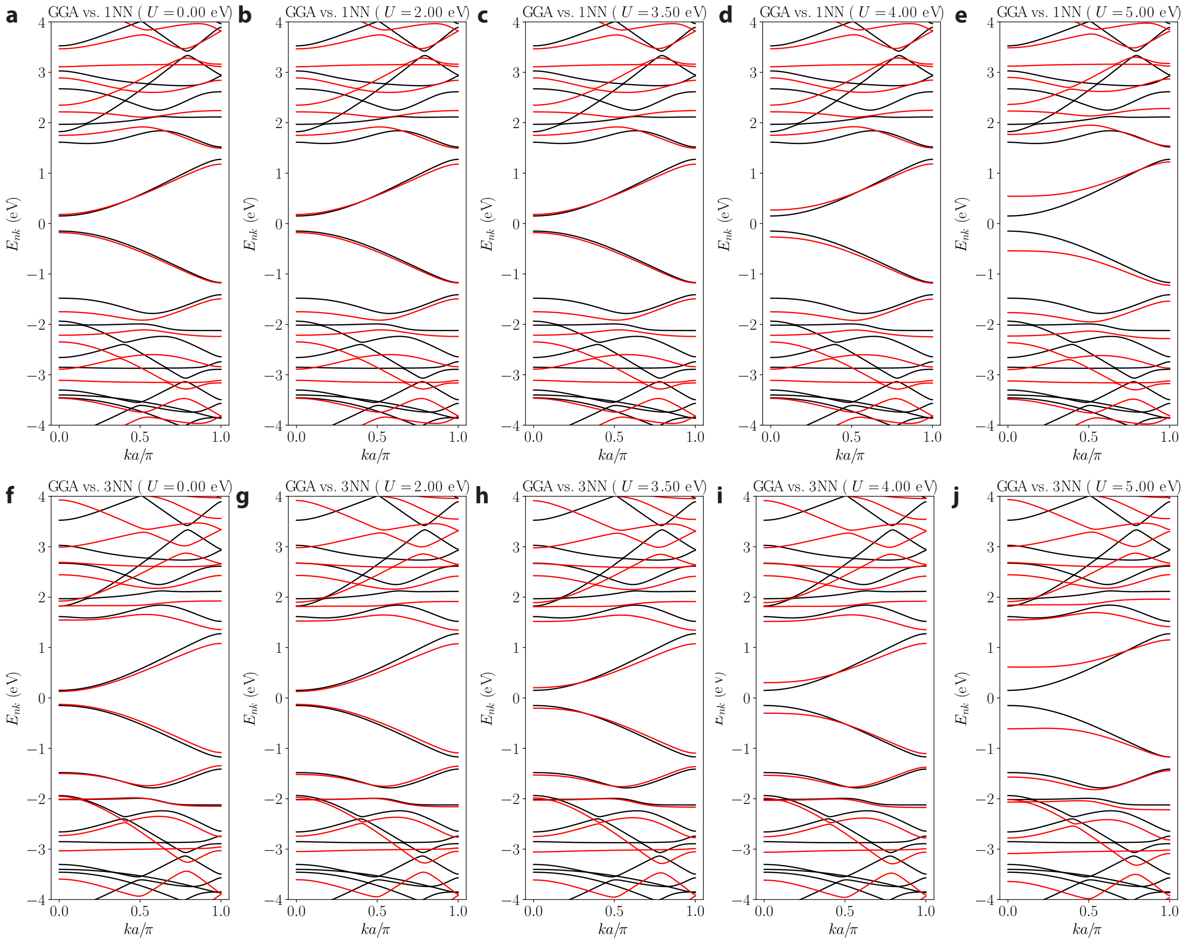

Supplementary Figure 9. Calculated 1D band structure for the (3,1)cGNR. We compare DFT-GGA (black lines) with the mean-field Hubbard model with different Coulomb repulsion U (red lines) within either (**a-e**) first-nearest neighbour couplings only ($t_1 = 2.70$ eV, top row) or (**f-j**) third-nearest neighbour couplings ($t_1 = 2.70$ eV, $t_2 = 0.20$ eV, and $t_3 = 0.18$ eV, bottom row).

Single-particle wave functions In Supplementary Figure 10 and Supplementary Figure 11 we analyze the eigenspectrum of energies and states in both the non-interacting ($U = 0$) and interacting ($U = 3.5$ eV) MFH Hamiltonians. The degree of spatial localization of each state is computed as $\eta_{\alpha\sigma} = \int dr|\psi_{\alpha\sigma}|^4$, also denoted the inverse participation ratio [17]. The HOMO/LUMO wave functions, shown in Supplementary Figure 12 for $U = 0$ and in Supplementary Figure 13 for $U = 3.5$ eV, are concentrated around the radical sites of the structure.

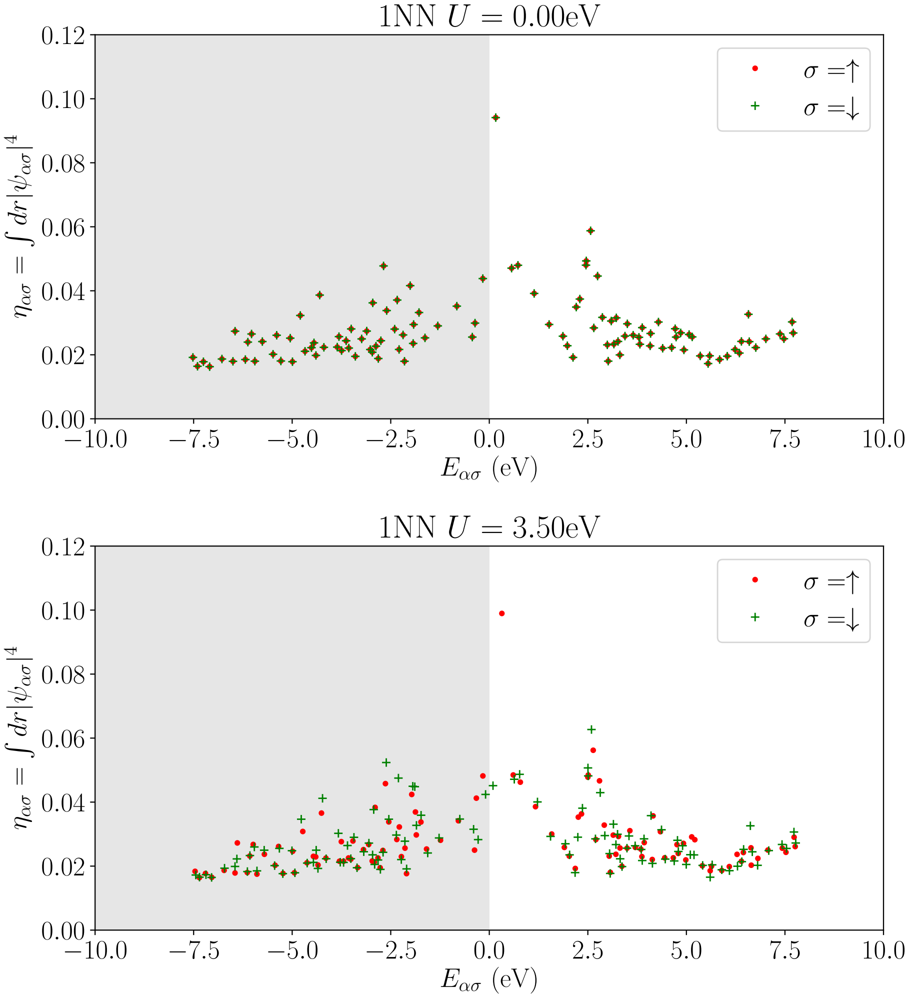

Supplementary Figure 10. Single-particle orbital localization $\eta_{\alpha\sigma}$ versus single-particle energy $E_{\alpha\sigma}$ in the 1NN model. Here we consider two characteristic values of U in both the 1NN and 3NN TB models. Among all states the LUMO orbital ($\sigma =\uparrow$ for finite U) is the most localized one.

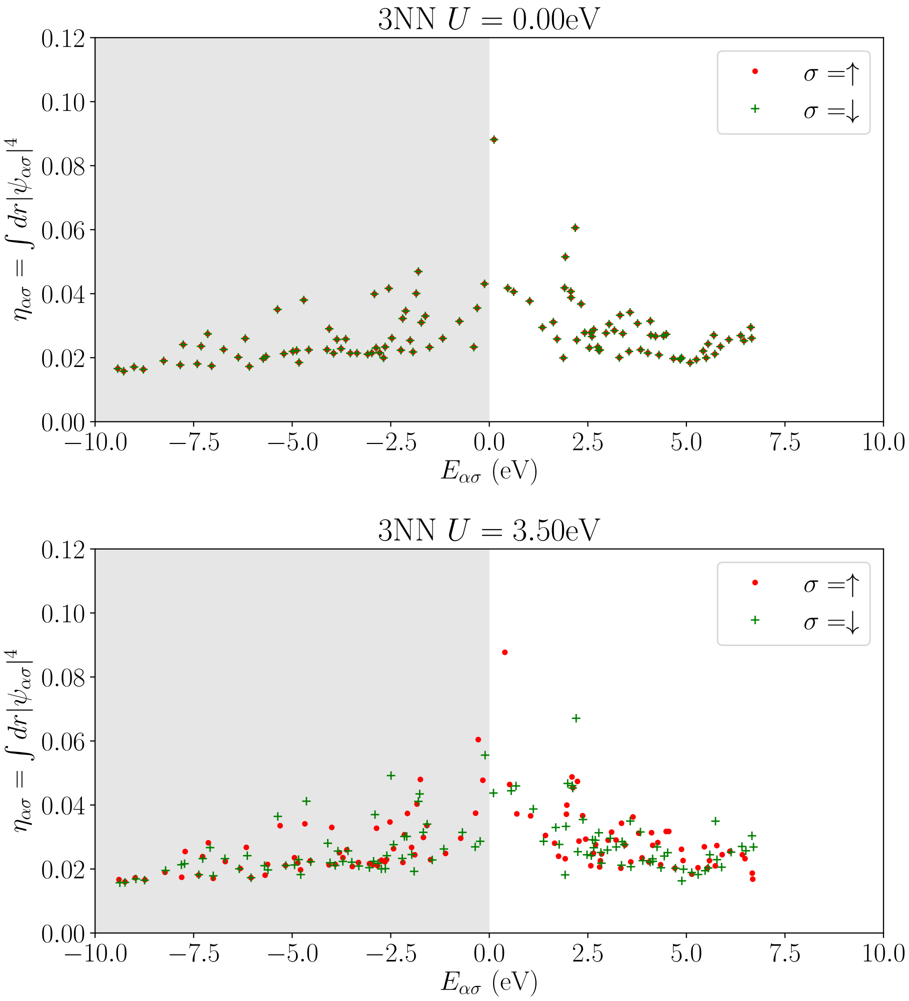

Supplementary Figure 11. Single-particle orbital localization $\eta_{\alpha\sigma}$ versus single-particle energy $E_{\alpha\sigma}$ in the 3NN model. Here we consider two characteristic values of U in both the 1NN and 3NN TB models. Among all states the LUMO orbital ($\sigma = \uparrow$ for finite U) is the most localized one.

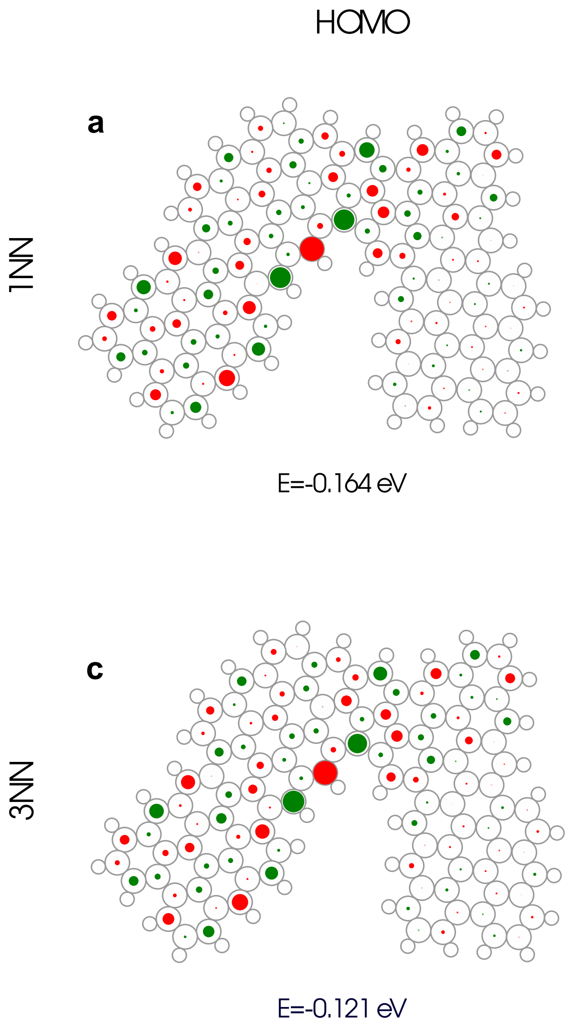

Supplementary Figure 12. Single-particle wave functions from MFH with $U = 0$ eV. a,b, Spatial distribution of the HOMO and LUMO wave functions within the 1NN model. c,d, Same as a,b but within the 3NN model. Panels c,d correspond to Fig. 3e in the main text. The single-particle energies relative to the midgap are stated below each plot. The size and color of the red-green circles reflect magnitude and phase of the wave function coefficients on each carbon atom, respectively.

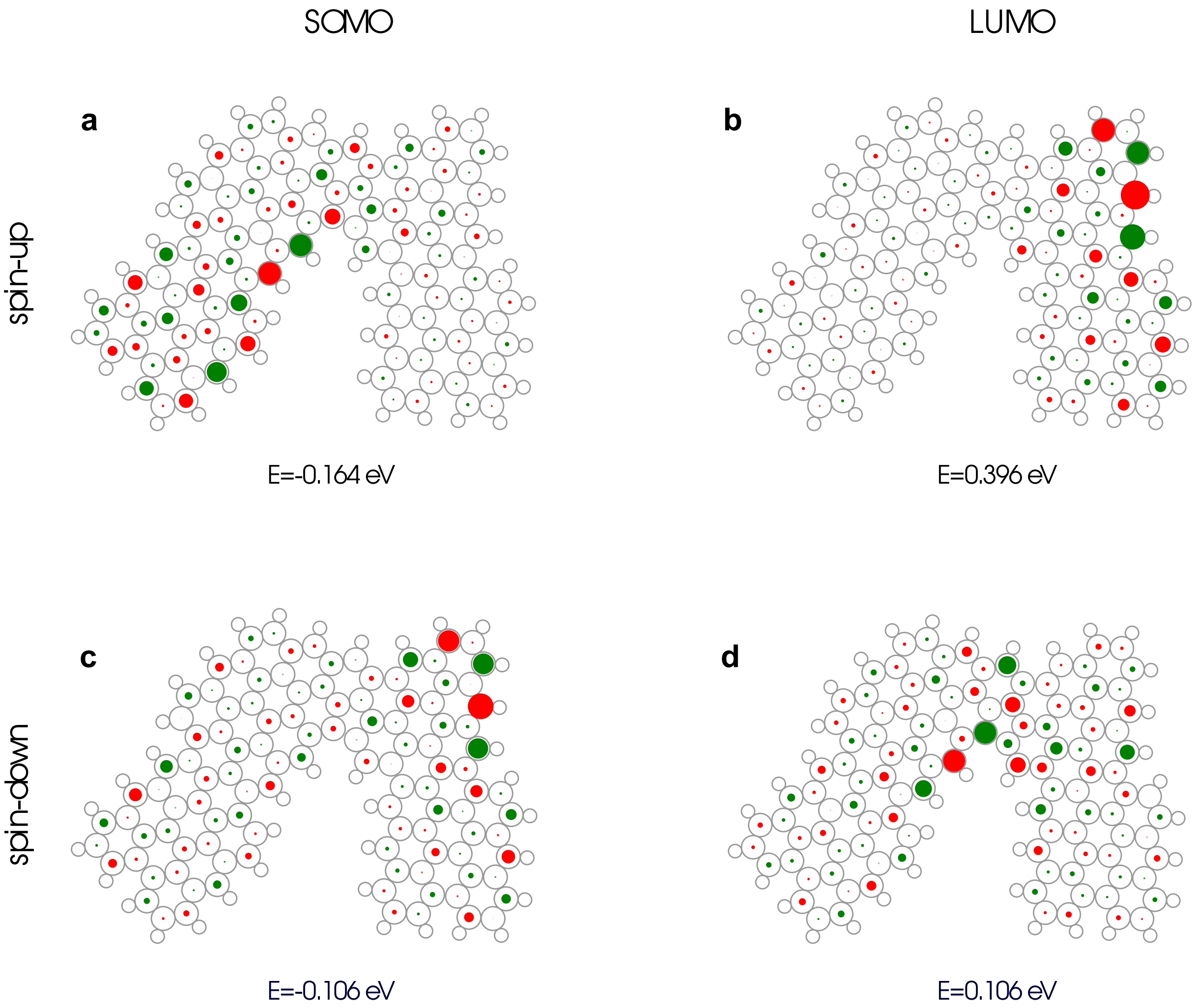

Supplementary Figure 13. Single-particle wave functions from MFH within the 3NN model with $U = 3.5$ eV. a,b, Spatial distribution of the SOMO and LUMO wave functions for the spin-up electrons, respectively. c,d, Same as a,b but for the spin-down electrons. The single-particle state energies relative to the midgap are stated below each plot. The size and color of the red-green circles reflect magnitude and phase of the wave function coefficients on each carbon atom, respectively.

Spin polarization In Supplementary Figure 14 we examine the spatial spin density obtained with MFH for both 1NN and 3NN models and varying on-site Coulomb repulsion. In the 1NN (3NN) model the onset of spin polarization occurs around $U = 3.1$ (2.7) eV. Compared with the DFT results in Supplementary Figure 6 (and the band structures of the previous section) we conclude that the 3NN model with Coulomb repulsion of the order $U = 3.5$ eV yields a very satisfactory agreement with DFT.

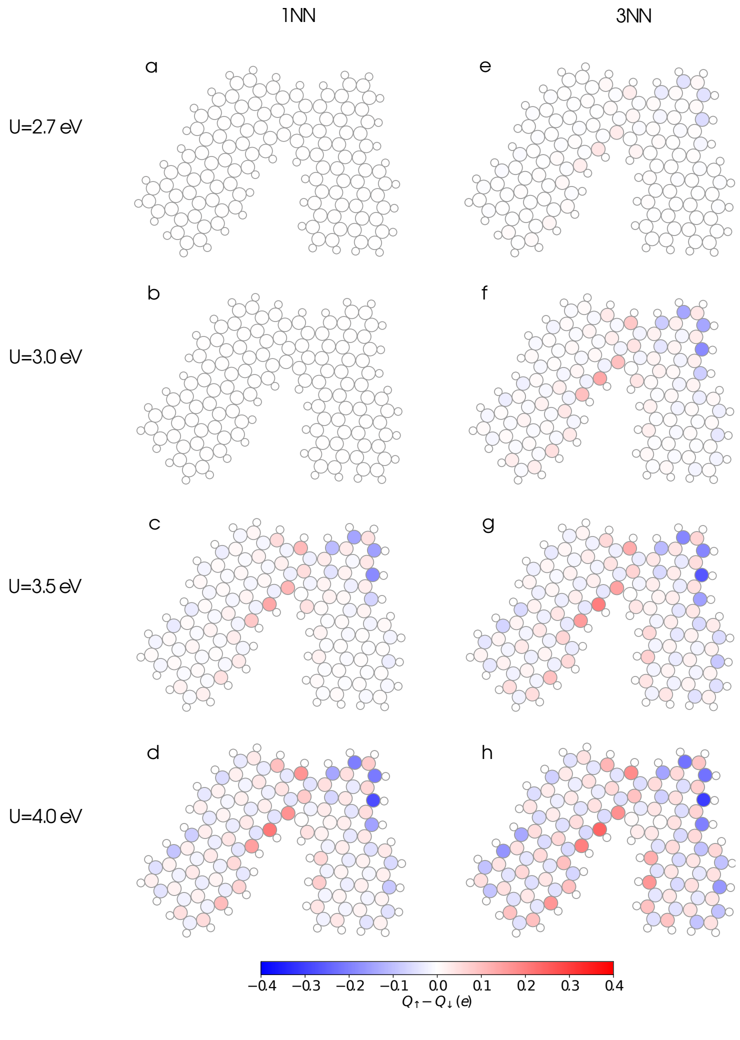

Supplementary Figure 14. Spin density from MFH calculations. (**a-d**) Spatial spin density, computed at site i as $Q_{i\uparrow} - Q_{i\downarrow}$, for different values of the on-site Coulomb repulsion parameter U. (**e-h**) Same as (a-d) but for the 3NN model.

Singlet-triplet excitations From Supplementary Figure 14 we have established that $U = 3.5$ eV yields a good description for these nanostructures as compared with DFT. As an approximation to the true singlet-triplet excitation energy J, we computed the mean-field energy difference ΔE_{ST} between the converged electronic configurations with $n_\uparrow = n_\downarrow$ and $n_\uparrow = n_\downarrow + 2$. In Supplementary Figure 15 we explore the variation of ΔE_{ST} with U for a Type 3 junction. Within the 3NN model a minimum is observed close to $\Delta E_{ST} \sim 19$ meV at $U \sim 3.5$ eV, in reasonable agreement with (albeit larger than) the experimentally observed peak splitting.

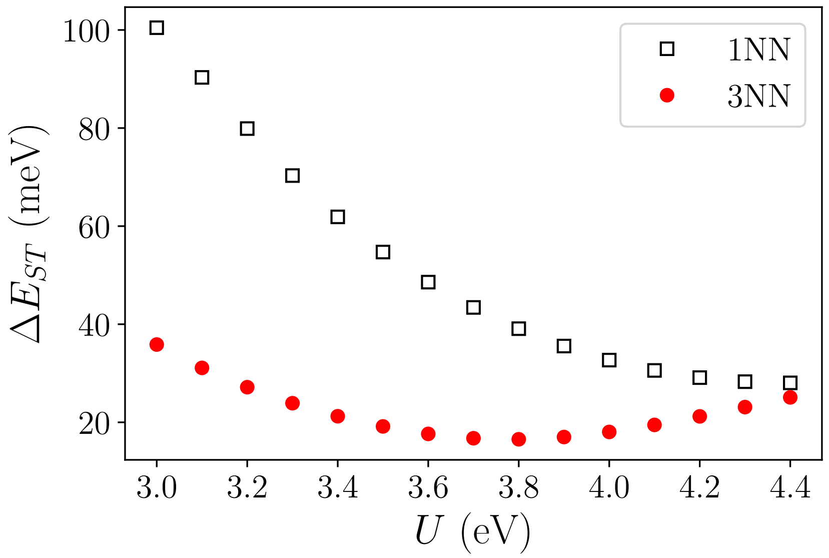

Supplementary Figure 15. Singlet-triplet excitation energy ΔE_{ST} versus U. We consider here the prototype molecule $(L, R) = (2, 2)$ within both the 1NN and 3NN models. The curves increase monotonically to the HOMO-LUMO gap $\Delta E_{\mathrm{H-L}} = 0.328$ (0.242) eV in the limit $U \to 0$ for the 1NN (3NN) model.

Supplementary Note 8: Singlet-triplet excitations vs molecular size

In the main text we have shown hat the singlet-triplet excitation energy of Type 3 junctions decreases with the size of the junctions, namely, with the length of the contacted ribbons. Here, we present results of the interaction energy between two coupled spins for several junctions with the structure shown in Supplementary Figure 16a, which corroborate this dependence.

In Supplementary Figure 16b we report experimentally extracted values of the singlet-triplet excitation energy from fits of a set of junctions, all with a similarly long arm a, as a function of the length of arm b. When the arm b consists of two precursor units, the singlet-triplet excitation energy is relatively low (below 3 meV), but for larger length of b (more than three precursor units) the energy difference raises quickly to around 8 meV. This trend is confirmed by the MFH simulations (Supplementary Figure 16c,d) both with the first-nearest hopping model (1NN) and the third-nearest hopping model (3NN). However, the experimental values are smaller than the computed ones. The origin of this could be related to the presence of a metal surface in the experiment, to the approximation of having only local Coulomb repulsion in the theory, or to limitation of the meanfield description.

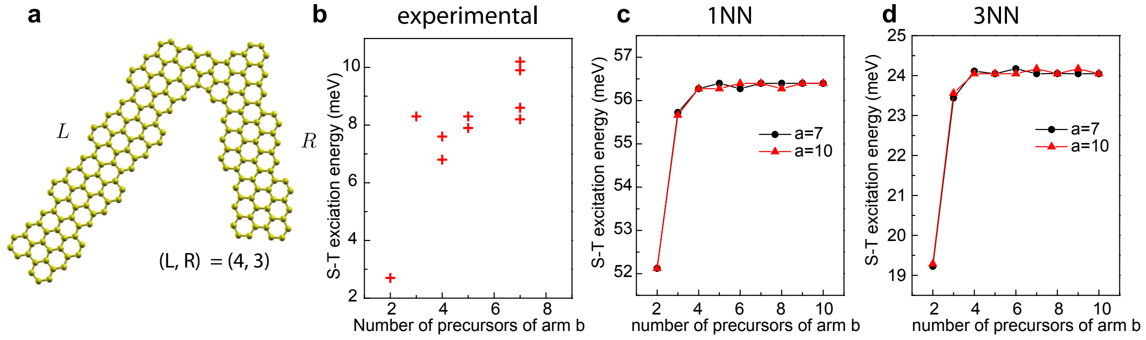

Supplementary Figure 16. Size-dependent singlet-triplet excitation energy of Type 3 junctions. a, Backbone structure of a Type 3 junction to illustrate how the size of a junction is measured in terms of the number of precursor units of each of its arms (defined as arm L and R as in the main text). b, Experimentally obtained spin-spin coupling energy J plotted as a function of the length of arm R. All junctions had arm $L > 7$. Each data point was extracted from a fit of dI/dV spectra as described in Fig. 2f. c, Calculated excitation energies as a function of the length of arm R within MFH with $U = 3.5$ eV in the first-nearest hopping model (1NN). Here the length of arm a are fixed as 7 and 10, respectively. d, Same as c but for the third-nearest hopping model (3NN).

Supplementary References

1. Talirz, L. *et al.* Termini of Bottom-Up Fabricated Graphene Nanoribbons. *J. Am. Chem. Soc.* **135**, 2060–2063 (2013).

2. Frota, H. O. Shape of the Kondo resonance. *Phys. Rev. B* **45**, 1096–1099 (1992).

3. Zhang, Y.-h. *et al.* Temperature and magnetic field dependence of a kondo system in the weak coupling regime. *Nat. Commun.* **4**, 2110 (2013).

4. Nagaoka, K., Jamneala, T., Grobis, M. & Crommie, M. F. Temperature Dependence of a Single Kondo Impurity. *Phys. Rev. Lett.* **88**, 77205 (2002).

5. Costi, T. A. Kondo effect in a magnetic field and the magnetoresistivity of kondo alloys. *Phys. Rev. Lett.* **85**, 1504–1507 (2000).

6. Solà, M. Forty years of Clar's aromatic π-sextet rule. *Front. Chem.* **1**, 22 (2013).

7. Soler, J. M. *et al.* The siesta method for ab initio order-n materials simulation. *J. Phys.: Condens. Matter* **14**, 2745–2779 (2002).

8. Ceperley, D. M. & Alder, B. J. Ground state of the electron gas by a stochastic method. *Phys. Rev. Lett.* **45**, 566–569 (1980).

9. Perdew, J. P., Burke, K. & Ernzerhof, M. Generalized gradient approximation made simple. *Phys. Rev. Lett.* **77**, 3865–3868 (1996).

10. Fernández-Rossier, J. & Palacios, J. J. Magnetism in Graphene Nanoislands. *Phys. Rev. Lett.* **99**, 177204 (2007).

11. Yazyev, O. V. Emergence of magnetism in graphene materials and nanostructures. *Rep. Prog. Phys.* **73**, 056501 (2010).

12. Yazyev, O. V., Capaz, R. B. & Louie, S. G. Theory of magnetic edge states in chiral graphene nanoribbons. *Phys. Rev. B* **84**, 115406 (2011).

13. Carvalho, A. R., Warnes, J. H. & Lewenkopf, C. H. Edge magnetization and local density of states in chiral graphene nanoribbons. *Phys. Rev. B* **89**, 245444 (2014).

14. Hancock, Y., Uppstu, A., Saloriutta, K., Harju, A. & Puska, M. J. Generalized tight-binding transport model for graphene nanoribbon-based systems. *Phys. Rev. B* **81**, 245402 (2010).

15. Papior, N. R. sisl: v0.9.2. doi: 10.5281/zenodo.597181 (2018).

16. Merino-Díez, N. *et al.* Unraveling the electronic structure of narrow atomically precise chiral graphene nanoribbons. *J. Phys. Chem. Lett.* **9**, 25–30 (2018).

17. Ortiz, R., García-Martínez, N. A., Lado, J. L. & Fernández-Rossier, J. Electrical spin manipulation in graphene nanostructures. *Phys. Rev. B* **97**, 195425 (2018).
